# Molecular diversity of *Annona* species and proximate fruit composition of selected genotypes

**DOI:** 10.1007/s13205-016-0520-9

**Published:** 2016-09-23

**Authors:** Hirdayesh Anuragi, Haresh L. Dhaduk, Sushil Kumar, Jitendra J. Dhruve, Mithil J. Parekh, Amar A. Sakure

**Affiliations:** 1 Department of Genetics and Plant Breeding, B. A. College of Agriculture, Anand Agricultural University, Anand, Gujarat 388 110 India; 2 Department of Agricultural Biotechnology, Centre of Excellence in Biotechnology, Anand Agricultural University, Anand, Gujarat 388 110 India; 3 Department of Biochemistry, B. A. College of Agriculture, Anand Agricultural University, Anand, Gujarat 388 110 India

**Keywords:** *Annona*, Fruit proximate composition, Genetic diversity, RAPD, SSR

## Abstract

Understanding the genetic variation in germplasm is of utmost importance for crop improvement. Therefore, efforts were made to analyse the molecular marker based genetic diversity of 20 *Annona* genotypes from five different species of family *Annonaceae*. During analysis, a set of 11 RAPD primers yielded a total of 152 bands with 80.01 % polymorphism and PIC for RAPD ranged from 0.86 to 0.92 with a mean of 0.89. With 93.05 % polymorphism, 12 SSR primers produced 39 amplicons. The PIC for SSRs ranged from 0.169 to 0.694 with of average of 0.339. The dendrogram produced from pooled molecular data of 11 RAPD and 12 SSR primers showed seven clusters at a cutoff value of 0.78. The dendrogram discriminated all the *Annona* genotypes suggesting that significant genetic diversity was present among the genotypes. Proximate fruit composition study of nine fruiting genotypes of *Annona* revealed that *A. squamosa* possessed significantly higher amount of most of studies biochemical which gives an opportunity to fruit breeders to improve the other *Annona* species. Likewise, *A. muricata* being rich in seed oil content can be exploited in oil industries.

**Electronic supplementary material:**

The online version of this article (doi:10.1007/s13205-016-0520-9) contains supplementary material, which is available to authorized users.

## Introduction

*Annona*, a member of family *Annonaceae,* is one of the largest tropical and subtropical families of trees, shrubs, and lianas with circa 135 genera and 2500 species distributed worldwide (Escribano et al. 2007). In genus *Annona*, merely six species, i.e. *A. squamosa* (widely cultivated), *A. reticulata, A. cherimola, A. muricata, A. atemoya* (a natural hybrid *A. squamosa* × *A. cherimola*) and *A. diversifolia* produce edible fruits. The origin of most of *Annona* species is South America and the Antilles, however, wild soursop (*A. muricata*) is thought to have originated in Africa (Pinto et al. 2005). The current distribution of important species covers almost all continents, with soursop and sugar apple showing the widest distribution, mainly in tropical regions. India is considered as secondary centre of origin for *A. squamosa*. Chromosome numbers of *Annona* are 2*n* = 2*x* = 14 and 16, except for *A. glabra*, which is a tetraploid species (2*n* = 4*x* = 28).

The pulp of *Annonas* is rich in minerals and vitamins (Gyamfi et al. 2011) and also a potential source of dietary fibre (up to 50 % w/w dry basis). High nutritive value of *A. cherimola* is due to fatty acids, edible fibres, carbohydrates, and minerals such as calcium, phosphorous and potassium (Lopez and Reginato 1990). *Annona* seeds, especially *A. squamosa* contain good amount of oil (Mariod et al. 2010) which can be exploited for industrial purpose. In addition, leaves, roots, barks, fruits and seeds of genus *Annona* have been considered as potential source of medicinally important compounds (Pinto et al. 2005). Hence, *Annonas*, a versatile plant with multiple use, are hardy and deciduous in nature, very easy to cultivate with minimum inputs, require comparatively little care and do not suffer from serious pests and diseases.

Fruits of *A. squamosa* and *A. muricata* occupy a promising position in today’s fruit market due to high demand by the processing industries (Santos et al. 2011). Though, their cultivation is still in incipient stages of domestication (Zonneveld et al. 2012). The genetic resources and plant diversity of outcrossing tropical tree species including *Annonas* are being eroded due to modernization of agriculture and land use changes. Hence, genetic resources of edible *Annonas* are exclusively present in situ, i.e. on farm, in home gardens/orchards and/or in natural populations.

Measuring genetic diversity is a mean for ranking the accessions for their use in breeding program. However, very few efforts have been carried out to identify diverse germplasm of *Annonas*. High variability among *Annona* population exists due to protogynous basis cross-pollination. In the past, morphological traits have been used as tools to characterize unexplored potential of germplasm for developing high yielding genotypes with better fruit quality (Folorunso and Modupe 2007). But traditional morphological markers are known to be significantly affected by edaphic and climatic conditions, hence, are not trustworthy due to high environmental influence (Kumar et al. 2014). Therefore, it is better to analyse diversity using molecular markers.

There are limited reports on exploitation of molecular markers for diversity analysis in *Annonas*. Few of these markers are random amplified polymorphic marker (RAPD) marker (Bharad et al. 2009; Ronning et al. 1995), amplified fragment length polymorphism (AFLP) markers (Rahman et al. 1998; Zhichang et al. 2011) and simple sequence repeat (SSR) markers (Escribano et al. 2004; Kwapata et al. 2007). On the other hand, information regarding nutritional value is of utmost important to select desired genotype for domestication in area of adaptation. Very little information is available on proximate analysis of *Annona* fruits (Onimawo 2002; Kulkarni et al. 2013; Boake et al. 2014). Keeping in view about scanty information of *Annonas*, efforts have been made to study the genetic diversity using RAPD and SSR molecular markers and proximate analysis of fruits of selected accessions.

## Materials and methods

### Plant materials and DNA isolation

**Fig. 1:**
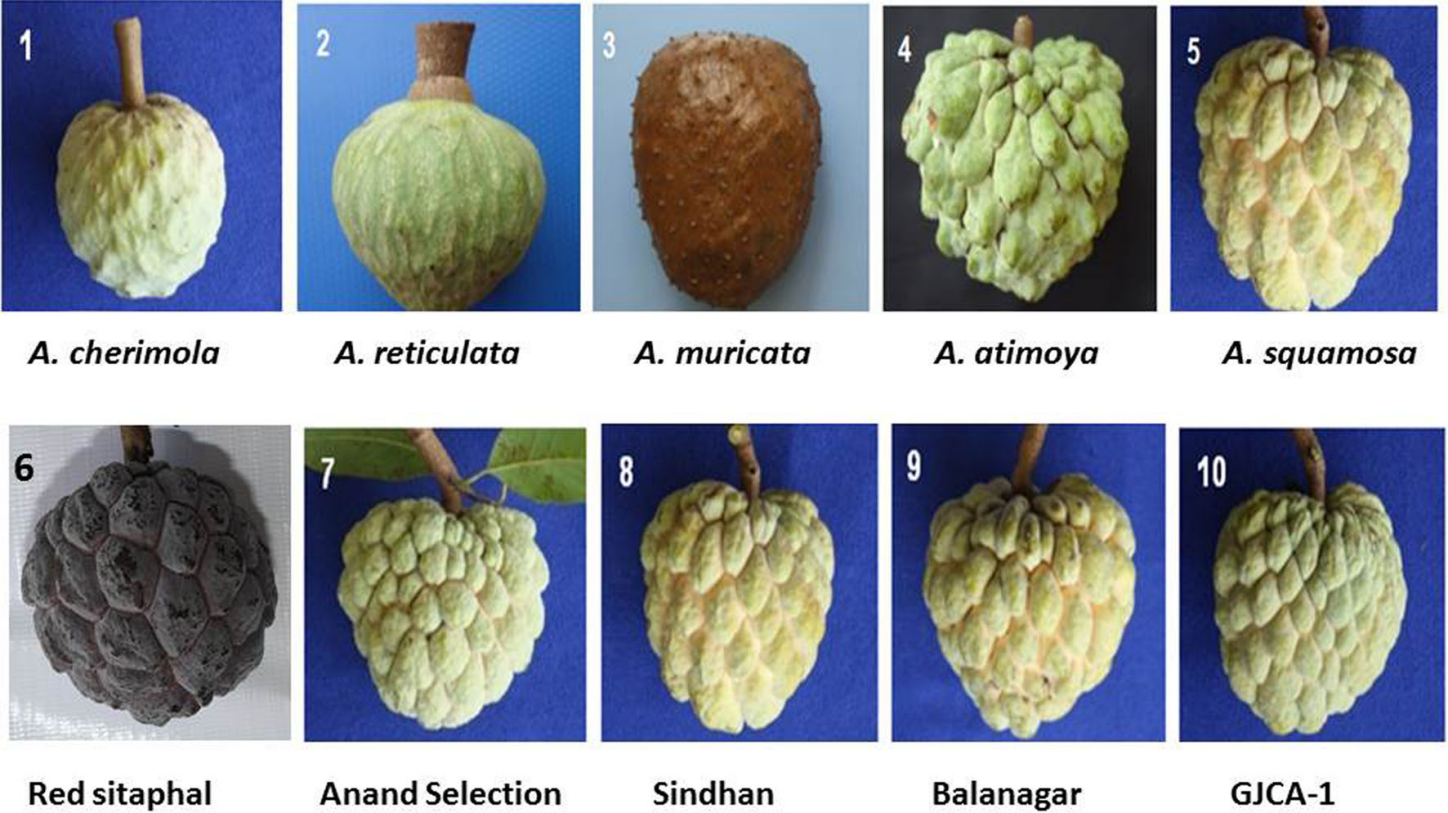
Fruit of different *Annona* species **1** *A. cherimola*; **2** *A. reticulata*; **3** *A. muricata*; **4** *A. atemoya*; **5** *A. squamosa* **6** Red Sitaphal; **7** Anand Selection; **8** Sindhan; **9** Balanagar and **10** GJCA-1

For molecular diversity analysis, a total of 20 *Annona* genotypes belonging to five different species were collected from various locations (Table 1; Fig. 1). DNA was extracted from young and tender leaves using CTAB method (Doyle and Doyle 1990). Extracted DNA was quantified using Nanodrop (Thermo scientific, USA) and further diluted to 20 ng/μl with TE buffer and stored at 4 °C for analysis.

**Table 1:** List of *Annona* accessions used in present study

Species	Accession	Collection area	Proximate analysis
*A. cherimola*	–	Horticulture farm, AAU, Anand	✓
*A. reticulata*	–	Horticulture farm, AAU, Anand	✓
*A. muricata*	–	Gir Forest, Gujarat	✓
*A. atemoya*	–	Horticulture farm, AAU, Anand	✓
*A. squamosa*	Red Sitaphal	Horticulture farm, AAU, Anand	✓
*A. squamosa*	Anand Selection	Horticulture farm, AAU, Anand	✓
*A. squamosa*	Sindhan	Horticulture farm, AAU, Anand	✓
*A. squamosa*	Balanagar	Horticulture farm, AAU, Anand	✓
*A. squamosa*	GJCA-1	Horticulture farm, AAU, Anand	✓
*A. squamosa*	Vidyanagar local	Anand, Gujarat	×
*A. squamosa*	ACC-1	Mumbai, Maharashtra	×
*A. squamosa*	ACC-2	Mumbai, Maharashtra	×
*A. squamosa*	ACC-3	Mumbai, Maharashtra	×
*A. squamosa*	ACC-4	Hyderabad, Telangana	×
*A. squamosa*	ACC-5	Hyderabad, Telangana	×
*A. squamosa*	ACC-6	Mumbai, Maharashtra	×
*A. squamosa*	Sindhan × Anand Selection	Horticulture farm, AAU, Anand	×
*A. squamosa*	Sindhan × Balanagar	Horticulture farm, AAU, Anand	×
*A. squamosa*	Anand Selection × Balanagar	Horticulture farm, AAU, Anand	×
*A. squamosa*	Balanagar × Red Sitaphal	Horticulture farm, AAU, Anand	×

### RAPD and SSR analysis

RAPD amplifications were carried out in 15 µl reaction volume containing: 40–50 ng DNA, 2 µl PCR Dream *Taq* buffer (10×) with 20 mM MgCl_2_ (Thermo Scientific, USA), 0.4 µl of 10 mM dNTPs (Thermo scientific, USA), 0.2 µl Dream *Taq* polymerase (5 U/μl, Thermo scientific, USA) and 1 μl of 10 pmol of primer (Table 2). Amplification was then performed in a DNA thermocycler (Eppendorf, Germany) using steps: (1) initial denaturation at 94 °C for 7 min, (2) 45 cycles of denaturation at 94 °C for 50 s, (3) primer annealing at 38 °C for 50 s, (4) extension at 72 °C for 1.2 min and (5) final extension at 72 °C for 7 min.

**Table 2:** Characterization of RAPD primers among different genotypes of *Annona* species

Locus name	Sequence (5′–3′)	Fragment size (bp)	Number of loci	Number of polymorphic loci	Polymorphism (%)	PIC
OPB-6	TGCTCTGCCC	423–1406	13	12	92.30	0.89
OPB-7	GGTGACGCAG	146–1322	19	18	94.73	0.92
OPB-8	GTCCACACGG	209–1328	16	14	87.50	0.89
OPB-10	CTGCTGGGAC	294–1166	15	10	66.67	0.92
OPB-12	CCTTGACGCA	246–1391	16	15	93.75	0.89
OPD-2	GGACCCAACC	311–1325	10	8	80.00	0.86
OPD-16	AGGGCGTAAG	180–1305	14	5	35.71	0.92
OPD-20	ACCCGGTCAC	302–1268	11	6	54.54	0.89
OPF-4	GGTGATCAGG	395–1308	12	10	83.33	0.86
OPF-5	CCGAATTCCC	404–1400	14	14	100.00	0.87
OPF-10	GGAAGCTTGG	451–1362	12	11	91.66	0.87
	Total		152	123	–	9.79
	Average		13.81	11.18	80.01	0.89

SSR amplification was carried out using 12 pairs of primers (Table 3) reported by Escribano et al. (2008). PCRs were performed in a 10 μl reaction mixture containing: 15 ng template DNA, 1 µl of PCR Dream *Taq* buffer (10×) with 20 mM MgCl_2_ (Thermo scientific, USA), 0.3 μl of each primer (10 pmol; forward and reverse), 0.3 μl of dNTPs (10 mM) and 0.15 µl Dream *Taq* polymerase (5U/μl, Thermo scientific, USA). The PCR cycling conditions were: 94 °C for 7 min as an initial denaturation step, followed by 35 cycles of 94 °C for 50 s, for 50 s at annealing temperature (48–57 °C depending upon the primer), 72 °C for 1 min (extension) and final extension at 72 °C for 7 min.

**Table 3:** Characterization of SSR primers among different genotypes of *Annona* species

Locus name	Sequence (5′–3′)	Fragment size (bp)	Number of alleles	Number of polymorphic alleles	Polymorphism (%)	PIC
LMCH-29	F: GTACCATCTTTTAGGAAATC	196–280	4	4	100	0.496
	R: TGCAATCTATGTTAGTCAC					
LMCH-43	F: CTAGTTCCAAGACGTGAGAGAT	210–375	3	3	100	0.177
	R: ATAGGAATAAGGGACTGTTGAG					
LMCH-48	F: TTAGAGTGAAAAGCGGCAAG	157–192	2	1	50	0.227
	R: TCAAGCTACAGAAAGTCTACCG					
LMCH-70	F: GAAGTTTTAGAGGCGATTCC	152–178	3	3	100	0.254
	R: TTTTGCCACTTTACTGTCAC					
LMCH-71	F: AGATAACACCCGCCCACTAT	282–497	3	2	66.67	0.169
	R: ACAACTTTTCTCCCAACCTATC					
LMCH-78	F: ATTTGATTGATTGATTTCCTA	172–235	3	3	100	0.265
	R: CTTTTGCTTTCTTTCACATC					
LMCH-79	F: GAAGCAAGTAGACACGTAGTA	212–384	5	5	100	0.694
	R: AGGGTTGGTATTTCTTTATAGT					
LMCH-112	F: TAACCCAGGATCTACAATAAT	194–278	4	4	100	0.525
	R: TTGCATACATTTTCCTATTT					
LMCH-114	F: AAAATGTAGTGTGAAAGATGAC	202–246	3	3	100	0.351
	R: GTCCATTCAGTTTTAAGTGC					
LMCH-119	F: CAGAAAATTAGCAGAGGACTCA	191–287	3	3	100	0.265
	R: GTGGGTTGGGTTTTTAGGTC					
LMCH-122	F: AGCAAAGATAAAGAGAAGATAA	190–212	3	3	100	0.310
	R: ATCCAAGCCTATTAACAACT					
LMCH-128	F: CTTGTTAAAATGGCTGTTACT	252–288	3	3	100	0.343
	R: GCATTGAGCTGACATAACTC					
	Total		39	37	–	4.068
	Average		3.25	3.08	93.05	0.339

Amplified products of RAPD and SSR were electrophoresed on 1.5 and 2.5 % agarose gel, respectively, using 1× TBE buffer. The gels were photographed by gel documentation system (Bio-Rad, Hercules, California). A 100-bp DNA ladder was used as a DNA size standard. Each experiment was repeated twice for each primer and only reproducible fingerprints (DNA bands) were considered for data analysis.

### Biochemical analysis

Out of 20 *Annona* genotypes, only nine were matured enough to produce fruits, hence proximate fruit composition analysis was performed only for nine genotypes. To determine various biochemical in fruits, three fully ripened mature fruits of similar and comparative stage were collected. A total of ten parameters of fruit and one parameter of seed, *i.e.* seed oil content was studied. For biochemical analysis, the fruit pulp and seeds was separated, freeze-dried, homogenised and stored at −20 °C until analysis. The starting material for biochemical analysis was 5 g pulp for moisture content, 2 g pulp each for ash and fibre content while 1 g pulp each for total carbohydrate, total soluble sugars (TSS), reducing sugars, protein, phenol, titratable acidity and ascorbic acid content. Similarly, 1 g seed powder (from homogenised composite seed sample) was used for oil content analysis. For proximate analysis, samples were prepared as per Onimawo (2002). Moisture content was obtained by heating the samples to a constant weight in a thermostatically controlled oven at 105 °C (Onimawo 2002). Total soluble sugar was estimated by the Anthrone method as suggested by Dubois et al. (1956). The ash, protein content, fibre content and total carbohydrates were obtained using the methods described by Association of Official Analytical Chemists (AOAC 1980). Reducing sugars were estimated by method of Nelson (1944) while phenol content was calculated by the method described by Bray and Thorpe (1954). Titratable acidity, ascorbic acid content and seed oil content were estimated as per Mehta et al. (1993).

### Data analysis

For each RAPD and SSR primer, the presence (1) or absence (0) of bands in each accession was scored to generate rectangular data matrix. The pairwise genetic similarity coefficient was calculated using Jaccard’ coefficient by NTSYS-pc v2.02 (Rohlf 1998). The matrices derived from RAPD and SSR data were correlated using MXCOMP module of NTSYS-pc. Polymorphism information content (PIC) was calculated according to Anderson et al. (1993). In case of biochemical parameters, data were subjected to evaluate arithmetic mean, standard deviation (SD), coefficient of variation (CV) using the standard formula specified in Chandel (1997).

## Results and discussion

### Molecular diversity and cluster analysis

#### RAPD analysis

**Fig. 2:**
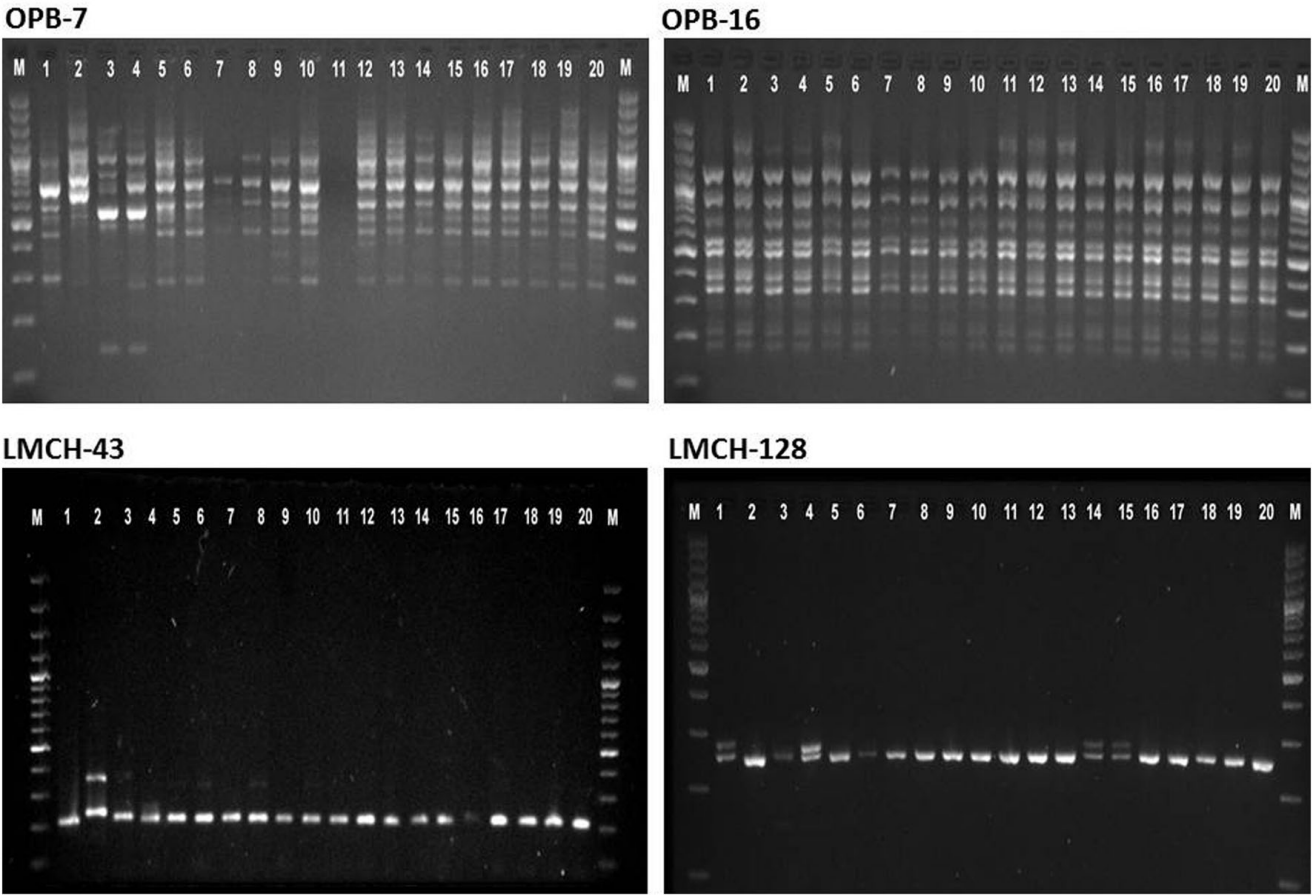
DNA amplification profile of RAPD (OPB-7 and OPB-16) and SSR (LMCH-29 and LMCH-79) markers

Eleven arbitrary decamer primers with clear and reproducible bands used for RAPD analysis could produce 152 fragments from the 20 genotypes of *Annonas*. The number of detected bands per primer varied from 10 to 19 with a mean of 13.81 bands per primer (Table 2; Fig. 2). Molecular size of the amplified PCR products ranged from 146 bp (OPB-7) to 1406 bp (OPB-6) which is comparable with results of Bharad et al. (2009) where fragment size ranged between 208 and 1354 bp. Out of total, 123 (80 %) loci were polymorphic. The number of polymorphic bands ranged from 5 to 18 with an average of 11.18. The highest polymorphism of 100 % was exhibited by primer OPF-5 while it was lowest (35.71 %) by OPD-16. The high polymorphism is an indication of prevalence of good diversity among accessions studied in present investigation. The polymorphism percentage, in this study, was much higher than the earlier reports in *Annonas* (73 %, Bharad et al. 2009; 29 %, Guimaraes et al. 2013). In contrast, Ronning et al. (1995) reported 93.5 % (86 out of 92 bands) polymorphism in *A. cherimola*, *A. squamosa* and their hybrids with 15 primers. Moreover, across the species polymorphism was 32 % higher than Elhawary et al. (2013) who showed merely 47.85 % polymorphism in four species (*A. cherimola, A. squamosa, A. muricata* and *A. glabra*) of the Annonaceae grown in Egypt.

The discrimination power of each RAPD primer was estimated by the PIC, which ranged from 0.86 (OPD-2 and OPF-4) to 0.92 (OPB-7, OPB-10 and OPD-16) with an average of 0.89 indicating the presence of high level of genetic diversity among different *Annona* genotypes. Genetic similarity matrix among cultivars showed an average distance ranging from 0.40 (*A. muricata*/ACC-4) to 0.93 (ACC-2/ACC-3) with a mean value of 0.70 (Table 2). The range of distance indicated moderate divergent in studied genotypes at the DNA level. This moderate diversity (7–60 %) range suggesting a good adaptability of *Annonas* in the region studied. The diversity recorded in present investigation was much higher than the earlier reported (19–57 % with AFLP) in China by Zhichang et al. (2011) in five *Annona* species.

#### SSR analysis

Microsatellites or SSRs, dispersed uniformly in genome, have become the markers of choice for fingerprinting purposes due to their high polymorphism, co-dominance nature, and reproducibility (Kumar et al. 2016). Development of SSRs is prohibitively expensive, but once the SSRs have been developed, they are cost- and time-effective in comparison to RAPD. Moreover, SSRs show cross genus- and species-amplification. Though, SSRs are potential marker to assess genetic diversity but have not been much exploited in *Annonas* especially *A. squamosa* (Gupta et al. 2015). Cross-species amplification of SSRs, developed in cherimoya, was successfully amplified in present study. This was is in congruence with Escribano et al. (2004) where SSRs markers of cherimoya were found highly transferable in different taxa of family Annonaceae. In this study, a total of 39 fragments were amplified from SSRs, of which 37 (93.05 %) were polymorphic (Table 3; Fig. 2). Lowest (50 %) polymorphism was produced by primer LMCH-48. The number of fragments per primer ranged from 2 to 5, with an average of 3.25 fragments per SSR; the number of polymorphic fragments ranged from 1 to 4, with an average of 3.08 fragments. The fragments per SSR was little lower than Escribano et al. (2007) where 4.9 bands/SSR were observed with 16 SSRs in *A. cherimola.* Though, it is higher than previous studies with isozymes (Perfectti and Pascual 1998). This indicates the effectiveness of SSRs to unravel diversity. In this study, amplicon size ranged from 152 bp (LMCH-70) to 497 bp (LMCH-71) which similar to results obtained previously in cherimoya with 15 SSRs (Escribano et al. 2004, 2008). PIC value ranged from 0.169 (LMCH-71) to 0.694 (LMCH-79) with an average of 0.339 indicating existence of sufficient genetic diversity among different *Annona* genotypes. With a mean of 0.67, similarity values among different *Annona* accessions ranged 0.12 (*A. reticulata* and *A. muricata*) to 1. A similar diversity level was observed in *A. cherimola* (Escribano et al. 2007) and in *A. senegalensis* (Kwapata et al. 2007).

#### Combined RAPD and SSR based cluster analysis

**Fig. 3:**
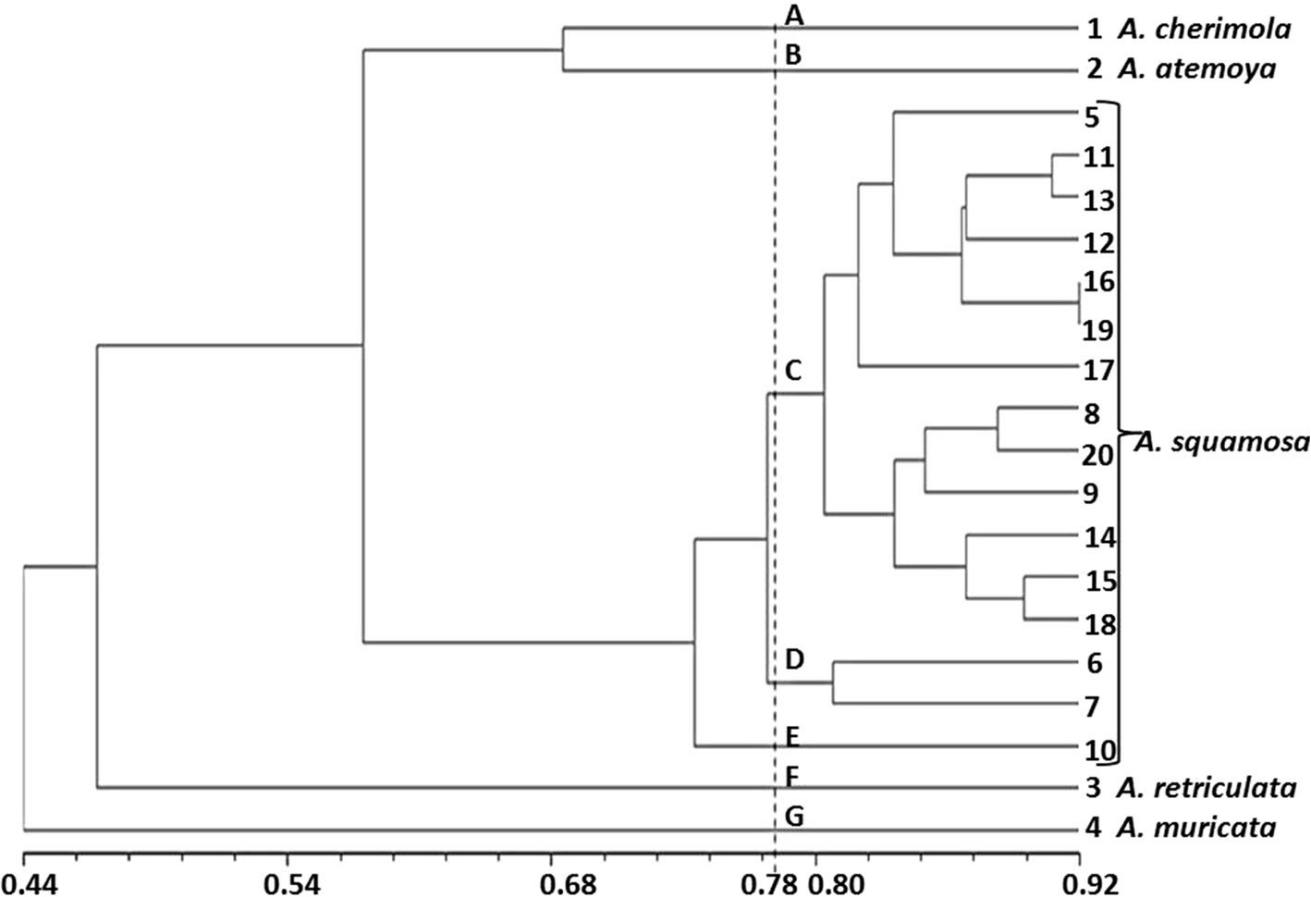
Dendrogram of 20 *Annona *genotypes based on combined RAPD and SSR data. (5. Red Sitaphal, 6. Anand Selection, 7. Sindhan, 8. Balanagar, 9. GJCA-1, 10. Vidyanagar Local, 11. ACC-1, 12. ACC-2, 13. ACC-3, 14. ACC-4, 15. ACC-5, 16. ACC-6, 17. Sindhan × Anand Selection, 18. Sindhan × Balanagar, 19. Anand Selection × Balanagar and 20. Balanagar × Red Sitaphal)

Matrices calculated for RAPD and SSR markers were compared via Mantel tests. Mantel test based correlation of similarity matrices was 0.86. Moreover, with the fact that RAPD and SSR target different portions of the genome, an UPGMA analysis was performed by combining both marker systems. This allowed a better coverage of genome. The dendrogram produced from pooled molecular data of 11 RAPD and 12 SSR primers showed seven clusters viz., cluster ‘A’ (*A. cherimola*), cluster ‘B’ (*A. atemoya*), cluster ‘C’ (Red Sitaphal, Balanagar, GJCA-1, ACC-1, ACC-2, ACC-3, ACC-4, ACC-5, ACC-6, Anand Selection × Balanagar, Sindhan × Anand Selection, Sindhan × Balanagar and Balanagar × Red Sitaphal), cluster ‘D’ (Anand Selection and Sindhan), cluster ‘E’ (Vidyanagar local), cluster ‘F’ (*A. reticulata*) and cluster ‘G’ (*A. muricata*) at cutoff value of 0.78 similarity coefficient (Supplementary Table 1; Fig. 2). Previously, Guimaraes et al. (2013) revealed five clusters with RAPD analysis of 64 *A. squamosa* accessions. In this study, atemoya was lying between its parental genome which is to be expected as atemoya, is the progeny of *A. cherimola* × *A. squamosa* hybridizations (Ronning et al. 1995). High level of genetic dissimilarity among *Annona* species as well as accessions of *A. squamosa* demonstrated that the level of genetic variation in the species is substantial and indicated that genetic base is quite broad. Hence, accessions can be exploited for *Annona* breeding for fruit yield and quality. The reason for high diversity can be attributed due to the collection of germplasm from various diverse locations (Fig. 3).

#### Fruit proximate composition

The nutrient composition of the fruits of different genotypes is presented in Table 4. *Annona* genotypes were found to be statistically different (*P* < 0.05) in the context of all studied fruit quality parameters. The minimum (72.55 %) and maximum (81.23 %) moisture content was observed from GJCA-1 and *A. muricata*, respectively. Fruits from all *Annona* species showed high moisture contents which is a typical characteristic of fleshy fruits. Low dry matter also indicated that the *Annonas* are more prone to spoilage and have low storability (Onyechi et al. 2012). The range of moisture content in this study is in accordance with previous reports where it ranged between 73.2 and 79.65 % in *A. squamosa* (Orsi et al. 2012; Othman et al. 2014) and 75–85.3 % in *A. muricata* (Orsi et al. 2012; Sawant and Dongre 2014).

**Table 4:** Statistics of fruit (pulp) quality parameters (fresh weight basis) and seed oil content (OC) of nine *Annona* genotypes [repetitions (*n*) = 2]

Genotypes	MC (%)	AC (%)	TC (%)	TSS (%)	RS (%)	FC (%)	PC (%)	PhC (%)	TA (%)	Asc (mg/100 g)	OC (%)
*A. cherimola*	79.26	1.56	17.54	14.41	4.33	2.22	1.69	0.35	0.36	19.60	29.33
*A. reticulata*	73.00	1.48	22.71	14.89	4.80	2.18	1.85	0.41	0.38	23.36	29.05
*A. muricata*	81.23	1.69	16.48	8.92	3.27	2.34	1.15	0.50	0.65	39.24	32.50
*A. atemoya*	76.63	1.62	20.71	14.62	6.67	3.82	1.84	0.40	0.37	32.74	28.41
*A. squamosa* (mean)	73.99	1.36	23.24	16.62	7.81	3.28	1.97	0.3	0.22	31.53	25.39
- Red Sitaphal	74.48	1.41	22.51	16.08	7.49	3.28	1.78	0.34	0.29	28.41	23.13
- Anand Selection	74.42	1.32	22.66	16.29	7.61	3.18	1.81	0.31	0.24	32.21	24.41
- Sindhan	73.65	1.43	23.04	16.69	7.81	3.38	2.13	0.26	0.23	32.06	26.56
- Balanagar	74.85	1.32	23.95	17.44	8.39	3.24	2.14	0.29	0.17	33.39	26.52
- GJCA-1	72.55	1.32	24.05	16.63	7.75	3.32	1.98	0.30	0.19	31.57	26.35
Minimum	72.55	1.32	16.48	8.92	3.27	2.18	1.15	0.26	0.17	19.60	23.13
Maximum	81.23	1.69	24.05	17.44	8.39	3.82	2.14	0.50	0.65	39.24	32.50
Mean	75.56	1.46	21.52	15.11	6.46	3.00	1.82	0.35	0.32	30.29	27.36
CD @ 5 %	5.37	0.14	2.16	1.31	0.48	0.38	0.34	0.05	0.05	3.50	3.10
SD	2.94	0.14	2.74	2.54	1.84	0.59	0.29	0.07	0.15	5.81	2.81
S.Em ±	1.79	0.05	0.72	0.44	0.16	0.13	0.11	0.02	0.02	1.17	1.03
CV (%)	4.11	5.67	5.80	5.02	4.30	7.35	10.72	8.86	9.59	6.67	6.55

*MC* moisture content,* AC* ash content,* TC* total carbohydrates,* TSS* total soluble sugars,* RS* reducing sugars,* FC* fibre content,* PC* protein content,* PhC* phenol content,* TA* titratable acidity* ASC* ascorbic acid content,* OC* seed oil content

Ash contents, an index of total mineral content, ranged from 1.32 % (Anand Selection, Balanagar and GJCA-1) to 1.69 % (*A. muricata*). A study from Andaman and Nicobar Islands, India by Singh et al. (2014) on *A. muricata* fruits also concluded that soursop pulp contained appreciable amount of ash (3.05 %).

The fruit has calories equivalent to that of mangoes (SCUC 2006) as the amount of sugars in fruit pulp of *A. squamosa* was found to be quite high (21.42 % of fresh pulp) (Sravanthi et al. 2014). The proximate analysis revealed that the main constituent of the fruits was total carbohydrates which were ranged from 16.48 % (*A. muricata*) to 24.05 % (GJCA-1) with mean value of 21.52 % indicating *Annonas* are excellent source of energy. The carbohydrate content has been reported to be 14.63–15.1 % in *A. muricata* pulp (Benero et al. 1971) and 19–25 % in *A. squamosa* (Duke and DuCellier 1993). The highest value of TSS, an index of fruit sweetness, was obtained from Balanagar (17.44 %) followed by Sindhan (16.69 %), GJCA-1 (16.63 %) and Anand Selection (16.29 %) while the lowest value (8.92 %) obtained from *A. muricata*. Analyses carried out in the United States reported *A. muricata* and *A. squamosa* fruit contained 13.54 g and 19.24 g (per 100 g edible portion, exclude seeds and skin) total sugars (http://www.bda-ieo.it/test/ComponentiAlimento.aspx?Lan=Eng&foodid=8634_2). Though, fruit with high TSS are very prone to splitting.

Reducing sugars are important for resistance mechanism against stresses and had the highest value of 8.39 % in Balanagar, while the lowest value of 3.27 % was obtained from *A. muricata*. Dietary fibre was recorded maximum for *A. atemoya* (3.82 %) and minimum for *A. reticulata* (2.18 %). Higher dietary fibre decreases the risk of many disorders, such as constipation, diabetes, cardiovascular diseases and obesity (Onimawo 2002).

Proteins are the building block of the body and are important components of various enzymes. Pinto et al. (2005) reported that pulp of *Annonas* have low protein but an appreciable vitamin C composition. In this study, protein content was found in the range of 1.15 (*A. muricata*) to 2.14 % (Balanagar). Previous reports on protein content in *A. squamosa* (1.2–2.4 %) by Pinto et al. (2005) and in *A. muricata* (1.21 %) by Amusa et al. (2003) are at par with present investigation.

Phenolic compounds are one of the most widely spread groups of phytochemicals. Plants need phenolic compounds for pigmentation, growth, reproduction, resistance to diseases and many other functions like antifeedants and attractants for pollinators, and functioning as sunscreen against UV light. In this study, it was found in the range of 0.26–0.50 % with maximum content for *A. muricata* and minimum for Sindhan. In addition to the antioxidant capacity, phenolics can influence the flavour determining fruit astringency and bitterness (Silva et al. 2013).

A variation between genotypes mean in connection to pulp titratable acidity which contributes to the acidity and aroma was highly significant. In current investigation, it ranged from 0.17 % in Balanagar to 0.65 % in *A. muricata*. The similar trend of titratable acidity was reported by Onimawo (2002) and Abbo et al. (2006) in *A. muricata*. Ascorbic acid, a potent antioxidant, was the range of 19.60 (*A. cherimola*) to 39.24 mg/100 g (*A. muricata*).

Very little attention has been paid to exploit the seeds for industrial purposes as little attempts have been made to extract oil from the seeds*. Annona* seeds were found rich enough in crude oil content, placing the seeds in the group of oil seeds, ranged from 23.13 % (Red Sitaphal) to 32.50 % (*A. muricata*). Findings with respect to oil studies are similar to the observations made by Djenontin et al. (2012). Though the seed oil content was lower than many oil seed crop like soybean, the result indicates that *Annona* oil can be new source of oil which can be exploited in industries instead of discarding as waste (Mariod et al. 2010).

## Conclusion

The conventional phenotypic based diversity analysis has now swapped by DNA markers. This study with RAPD and SSR markers suggested that at DNA level there is considerable genetic diversity among *Annona* genotypes. Similarly, *Annona* genotypes were significantly diverse for investigated biochemical parameters. Interestingly, *A. muricata*, a rich source of seed oil, can be exploited in oil industries. The genotypes characterized based on molecular marker and biochemical gives an opportunity to fruit breeders to alter the fruit qualities of genus *Annona*.

## Electronic supplementary material

Below is the link to the electronic supplementary material.

Supplementary material 1 (DOCX 46 kb)
