# Supplementary material for: Molecular diversity of Annona species and proximate fruit composition of selected genotypes
Source: 3 Biotech. 2016 Sep 23;6(2):204. doi: 10.1007/s13205-016-0520-9 (PMC5035287; doi:10.1007/s13205-016-0520-9)
Supplement: Supplementary file 1 — Supplementary material 1 (DOCX 46 kb) [file 13205_2016_520_MOESM1_ESM.docx]

Supplementary Table 1 Jaccard’s similarity coefficient based on 11 RAPD and 12 SSR markers used for diversity analysis of 20 *Annona* genotypes

| Accession | | 1 | 2 | 3 | 4 | 5 | 6 | 7 | 8 | 9 | 10 | 11 | 12 | 13 | 14 | 15 | 16 | 17 | 18 | 19 |
| --- | --- | --- | --- | --- | --- | --- | --- | --- | --- | --- | --- | --- | --- | --- | --- | --- | --- | --- | --- | --- |
| RAPD | 2 | 0.5 | 1 |  |  |  |  |  |  |  |  |  |  |  |  |  |  |  |  |  |
| SSR | 2 | 0.29 | 1 |  |  |  |  |  |  |  |  |  |  |  |  |  |  |  |  |  |
| RAPD+SSR | 2 | 0.46 | 1 |  |  |  |  |  |  |  |  |  |  |  |  |  |  |  |  |  |
| RAPD | 3 | 0.42 | 0.45 | 1 |  |  |  |  |  |  |  |  |  |  |  |  |  |  |  |  |
| SSR | 3 | 0.39 | 0.12 | 1 |  |  |  |  |  |  |  |  |  |  |  |  |  |  |  |  |
| RAPD+SSR | 3 | 0.42 | 0.39 | 1 |  |  |  |  |  |  |  |  |  |  |  |  |  |  |  |  |
| RAPD | 4 | 0.68 | 0.49 | 0.53 | 1 |  |  |  |  |  |  |  |  |  |  |  |  |  |  |  |
| SSR | 4 | 0.69 | 0.25 | 0.37 | 1 |  |  |  |  |  |  |  |  |  |  |  |  |  |  |  |
| RAPD+SSR | 4 | 0.68 | 0.44 | 0.5 | 1 |  |  |  |  |  |  |  |  |  |  |  |  |  |  |  |
| RAPD | 5 | 0.63 | 0.5 | 0.42 | 0.65 | 1 |  |  |  |  |  |  |  |  |  |  |  |  |  |  |
| SSR | 5 | 0.5 | 0.21 | 0.47 | 0.45 | 1 |  |  |  |  |  |  |  |  |  |  |  |  |  |  |
| RAPD+SSR | 5 | 0.61 | 0.45 | 0.43 | 0.57 | 1 |  |  |  |  |  |  |  |  |  |  |  |  |  |  |
| RAPD | 6 | 0.57 | 0.5 | 0.45 | 0.58 | 0.83 | 1 |  |  |  |  |  |  |  |  |  |  |  |  |  |
| SSR | 6 | 0.45 | 0.22 | 0.33 | 0.37 | 0.79 | 1 |  |  |  |  |  |  |  |  |  |  |  |  |  |
| RAPD+SSR | 6 | 0.55 | 0.46 | 0.43 | 0.54 | 0.82 | 1 |  |  |  |  |  |  |  |  |  |  |  |  |  |
| RAPD | 7 | 0.59 | 0.45 | 0.42 | 0.56 | 0.78 | 0.81 | 1 |  |  |  |  |  |  |  |  |  |  |  |  |
| SSR | 7 | 0.5 | 0.21 | 0.47 | 0.45 | 1 | 0.79 | 1 |  |  |  |  |  |  |  |  |  |  |  |  |
| RAPD+SSR | 7 | 0.58 | 0.41 | 0.43 | 0.54 | 0.81 | 0.81 | 1 |  |  |  |  |  |  |  |  |  |  |  |  |
| RAPD | 8 | 0.64 | 0.52 | 0.45 | 0.6 | 0.75 | 0.75 | 0.81 | 1 |  |  |  |  |  |  |  |  |  |  |  |
| SSR | 8 | 0.43 | 0.21 | 0.32 | 0.35 | 0.73 | 0.92 | 0.73 | 1 |  |  |  |  |  |  |  |  |  |  |  |
| RAPD+SSR | 8 | 0.6 | 0.47 | 0.44 | 0.55 | 0.75 | 0.78 | 0.8 | 1 |  |  |  |  |  |  |  |  |  |  |  |
| RAPD | 9 | 0.68 | 0.5 | 0.45 | 0.65 | 0.8 | 0.79 | 0.74 | 0.81 | 1 |  |  |  |  |  |  |  |  |  |  |
| SSR | 9 | 0.45 | 0.22 | 0.33 | 0.37 | 0.79 | 1 | 0.79 | 0.92 | 1 |  |  |  |  |  |  |  |  |  |  |
| RAPD+SSR | 9 | 0.64 | 0.46 | 0.44 | 0.59 | 0.8 | 0.82 | 0.75 | 0.83 | 1 |  |  |  |  |  |  |  |  |  |  |
| RAPD | 10 | 0.62 | 0.49 | 0.44 | 0.64 | 0.79 | 0.75 | 0.67 | 0.7 | 0.83 | 1 |  |  |  |  |  |  |  |  |  |
| SSR | 10 | 0.61 | 0.18 | 0.53 | 0.59 | 0.76 | 0.61 | 0.76 | 0.58 | 0.61 | 1 |  |  |  |  |  |  |  |  |  |
| RAPD+SSR | 10 | 0.62 | 0.43 | 0.45 | 0.64 | 0.79 | 0.73 | 0.69 | 0.68 | 0.8 | 1 |  |  |  |  |  |  |  |  |  |
| RAPD | 11 | 0.65 | 0.56 | 0.46 | 0.66 | 0.88 | 0.81 | 0.72 | 0.79 | 0.87 | 0.83 | 1 |  |  |  |  |  |  |  |  |
| SSR | 11 | 0.45 | 0.22 | 0.33 | 0.37 | 0.79 | 1 | 0.79 | 0.92 | 1 | 0.61 | 1 |  |  |  |  |  |  |  |  |
| RAPD+SSR | 11 | 0.62 | 0.5 | 0.45 | 0.6 | 0.87 | 0.83 | 0.73 | 0.81 | 0.89 | 0.8 | 1 |  |  |  |  |  |  |  |  |
| RAPD | 12 | 0.61 | 0.59 | 0.45 | 0.61 | 0.78 | 0.77 | 0.71 | 0.77 | 0.75 | 0.74 | 0.84 | 1 |  |  |  |  |  |  |  |
| SSR | 12 | 0.48 | 0.2 | 0.44 | 0.48 | 0.93 | 0.73 | 0.93 | 0.69 | 0.73 | 0.82 | 0.73 | 1 |  |  |  |  |  |  |  |
| RAPD+SSR | 12 | 0.6 | 0.52 | 0.45 | 0.59 | 0.81 | 0.77 | 0.74 | 0.77 | 0.75 | 0.75 | 0.83 | 1 |  |  |  |  |  |  |  |
| RAPD | 13 | 0.64 | 0.58 | 0.45 | 0.64 | 0.81 | 0.76 | 0.72 | 0.78 | 0.77 | 0.76 | 0.89 | 0.93 | 1 |  |  |  |  |  |  |
| SSR | 13 | 0.45 | 0.22 | 0.33 | 0.37 | 0.79 | 1 | 0.79 | 0.92 | 1 | 0.61 | 1 | 0.73 | 1 |  |  |  |  |  |  |
| RAPD+SSR | 13 | 0.61 | 0.52 | 0.44 | 0.59 | 0.81 | 0.79 | 0.73 | 0.8 | 0.8 | 0.74 | 0.91 | 0.9 | 1 |  |  |  |  |  |  |
| RAPD | 14 | 0.67 | 0.49 | 0.4 | 0.59 | 0.77 | 0.76 | 0.73 | 0.8 | 0.82 | 0.76 | 0.78 | 0.78 | 0.77 | 1 |  |  |  |  |  |
| SSR | 14 | 0.57 | 0.21 | 0.39 | 0.45 | 0.86 | 0.79 | 0.86 | 0.73 | 0.79 | 0.67 | 0.79 | 0.8 | 0.79 | 1 |  |  |  |  |  |
| RAPD+SSR | 14 | 0.66 | 0.44 | 0.4 | 0.56 | 0.79 | 0.77 | 0.75 | 0.8 | 0.82 | 0.75 | 0.78 | 0.79 | 0.77 | 1 |  |  |  |  |  |
| RAPD | 15 | 0.67 | 0.53 | 0.45 | 0.64 | 0.84 | 0.81 | 0.76 | 0.85 | 0.84 | 0.77 | 0.86 | 0.82 | 0.83 | 0.88 | 1 |  |  |  |  |
| SSR | 15 | 0.5 | 0.21 | 0.32 | 0.4 | 0.73 | 0.92 | 0.73 | 0.86 | 0.92 | 0.58 | 0.92 | 0.69 | 0.92 | 0.86 | 1 |  |  |  |  |
| RAPD+SSR | 15 | 0.64 | 0.48 | 0.44 | 0.59 | 0.83 | 0.83 | 0.76 | 0.85 | 0.86 | 0.74 | 0.87 | 0.81 | 0.85 | 0.88 | 1 |  |  |  |  |
| RAPD | 16 | 0.6 | 0.54 | 0.45 | 0.65 | 0.83 | 0.8 | 0.7 | 0.77 | 0.74 | 0.77 | 0.85 | 0.91 | 0.88 | 0.77 | 0.83 | 1 |  |  |  |
| SSR | 16 | 0.45 | 0.22 | 0.33 | 0.37 | 0.79 | 1 | 0.79 | 0.92 | 1 | 0.61 | 1 | 0.73 | 1 | 0.79 | 0.92 | 1 |  |  |  |
| RAPD+SSR | 16 | 0.59 | 0.49 | 0.44 | 0.59 | 0.83 | 0.83 | 0.72 | 0.79 | 0.77 | 0.75 | 0.87 | 0.89 | 0.89 | 0.78 | 0.85 | 1 |  |  |  |
| RAPD | 17 | 0.58 | 0.53 | 0.45 | 0.61 | 0.76 | 0.75 | 0.65 | 0.68 | 0.69 | 0.7 | 0.78 | 0.81 | 0.79 | 0.7 | 0.74 | 0.84 | 1 |  |  |
| SSR | 17 | 0.45 | 0.22 | 0.33 | 0.37 | 0.79 | 1 | 0.79 | 0.92 | 1 | 0.61 | 1 | 0.73 | 1 | 0.79 | 0.92 | 1 | 1 |  |  |
| RAPD+SSR | 17 | 0.57 | 0.48 | 0.44 | 0.56 | 0.77 | 0.78 | 0.67 | 0.71 | 0.73 | 0.69 | 0.81 | 0.81 | 0.81 | 0.72 | 0.77 | 0.86 | 1 |  |  |
| RAPD | 18 | 0.61 | 0.52 | 0.44 | 0.62 | 0.84 | 0.79 | 0.76 | 0.79 | 0.78 | 0.79 | 0.82 | 0.84 | 0.81 | 0.86 | 0.88 | 0.87 | 0.75 | 1 |  |
| SSR | 18 | 0.45 | 0.22 | 0.33 | 0.37 | 0.79 | 1 | 0.79 | 0.92 | 1 | 0.61 | 1 | 0.73 | 1 | 0.79 | 0.92 | 1 | 1 | 1 |  |
| RAPD+SSR | 18 | 0.6 | 0.47 | 0.43 | 0.57 | 0.84 | 0.82 | 0.77 | 0.81 | 0.81 | 0.77 | 0.85 | 0.83 | 0.84 | 0.85 | 0.89 | 0.89 | 0.78 | 1 |  |
| RAPD | 19 | 0.61 | 0.54 | 0.44 | 0.62 | 0.86 | 0.77 | 0.7 | 0.73 | 0.74 | 0.75 | 0.84 | 0.86 | 0.81 | 0.77 | 0.8 | 0.9 | 0.83 | 0.86 | 1 |
| SSR | 19 | 0.45 | 0.22 | 0.33 | 0.37 | 0.79 | 1 | 0.79 | 0.92 | 1 | 0.61 | 1 | 0.73 | 1 | 0.79 | 0.92 | 1 | 1 | 1 | 1 |
| RAPD+SSR | 19 | 0.59 | 0.49 | 0.43 | 0.57 | 0.85 | 0.8 | 0.72 | 0.76 | 0.78 | 0.73 | 0.86 | 0.84 | 0.83 | 0.78 | 0.82 | 0.92 | 0.85 | 0.88 | 1 |
| RAPD | 20 | 0.67 | 0.53 | 0.45 | 0.64 | 0.80 | 0.79 | 0.78 | 0.87 | 0.84 | 0.75 | 0.82 | 0.8 | 0.81 | 0.82 | 0.86 | 0.81 | 0.73 | 0.84 | 0.78 |
| SSR | 20 | 0.45 | 0.22 | 0.33 | 0.37 | 0.79 | 1 | 0.79 | 0.92 | 1 | 0.61 | 1 | 0.73 | 1 | 0.79 | 0.92 | 1 | 1 | 1 | 1 |
| RAPD+SSR | 20 | 0.63 | 0.48 | 0.44 | 0.59 | 0.8 | 0.82 | 0.78 | 0.88 | 0.87 | 0.73 | 0.85 | 0.8 | 0.84 | 0.82 | 0.87 | 0.84 | 0.76 | 0.87 | 0.81 |

*1-A. cherimola*; 2- *A. reticulata*; 3- *A. muricata*; 4- *A. atemoya*; 5- Red Sitaphal; 6- Anand Selection; 7- Sindhan; 8- Balanagar; 9- GJCA-1; 10- Vidyanagar local; 11- ACC-1; 12- ACC-2; 13- ACC-3; 14- ACC-4; 15- ACC-5; 16- ACC-6; 17- Sindhan×Anand selection; 18- Sindhan×Balanagar; 19- Anand selection×Balanagar and 20- Balanagar×Red Sitaphal.
